# Capturing yeast associated with grapes and spontaneous fermentations of the Negro Saurí minority variety from an experimental vineyard near León

**DOI:** 10.1038/s41598-021-83123-1

**Published:** 2021-02-12

**Authors:** Isora González-Alonso, Michelle Elisabeth Walker, María-Eva Vallejo-Pascual, Gérmán Naharro-Carrasco, Vladimir Jiranek

**Affiliations:** 1 Department of Animal Health, Universidad de León, Campus de Vegazana, León, Spain; 2 Department of Wine Science, The University of Adelaide, Waite Campus, Urrbrae, SA 5064 Australia; 3 Department of Economy and Statistics, Universidad de León, Campus de Vegazana, León, Spain; 4 Australian Research Council Training Centre for Innovative Wine Production, Adelaide, Australia

**Keywords:** Ecology, Microbiology

## Abstract

‘Microbial terroir’ relates to the influence of autochthonous yeasts associated with a grape cultivar on the resultant wine. Geographic region, vineyard site and topography, climate and vintage influence the biodiversity of these microbial communities. Current research focus attempts to correlate their ‘microbial fingerprint’ to the sensorial and chemical characteristics of varietal wines from distinct geographical wine regions. This study focuses on the minor red grape variety, Negro Saurí, which has seen a resurgence in the León Appellation of Origin in Spain as a varietal wine. An experimental vineyard at Melgarajo S.A. (42° 15′ 48.68_N 5° 9′ 56.66_W) was sampled over four consecutive vintages, with autochthonous yeasts being isolated from grapes, must and pilot-scale un-inoculated fermentations, and identified by ITS sequencing. Forty-nine isolates belonging to *Metschnikowia pulcherrima*, *Lachancea thermotolerans*, *Hanseniaspora uvarum* and *Torulaspora delbrueckii* were isolated from grapes and must, and early stages of fermentation dependent on seasonal variation. *Saccharomyces cerevisiae* predominated throughout fermentation, as a heterogeneous and dynamic population, with seven major biotypes identified amongst 110 isolates across four consecutive vintages. Twenty-four *S. cerevisiae* isolates representing five strains dominated in two or more vintages. Their persistence through fermentation warrants further validation of their oenological properties as starter cultures.

## Introduction

Autochthonous yeast, indigenous to a given wine region provide an opportunity to enhance wine, with the resulting style and organoleptic characteristics corresponding to a particular geographical 'terroir'^1,2^. These microbial communities are geographically distributed; each area having a characteristic population of microorganisms, which vary over time and have their own dynamics according to different aspects related to cultivar, vineyard management and climate^3^. Such populations are being defined as a ‘microbiological fingerprint’ of the region^4^, and are affected by the geographical location, grape variety, and vine development (reviewed in^5^).

*Aureobasidium pullulans* and *Metschnikowia*, *Hanseniaspora* (*Kloeckera*), *Cryptococcus* and *Rhodotorula* species predominate on healthy grape berries at different stages of maturity (reviewed by^6^). *Saccharomyces cerevisiae* is the principal yeast during fermentation^3,7^ and often is impossible to isolate from healthy, mature grapes^8,9^ indicating the winemaking environment as a reservoir. The vineyard and winery microbiota form the inoculum for ‘spontaneous’ wine fermentations. A specific succession of yeast communities^10^ occurs, with *Hanseniaspora*, *Candida*, *Metschnikowia*, *Pichia*, *Issatchenkia* and *Kluyveromyces* species often present in the initial stages^11^, *Saccharomyces* outcompete later in fermentation. Research has focused on the application of non-*Saccharomyces* yeasts to winemaking, given their ability to produce enzymes of biotechnological value^12,13^, and volatile and non-volatile constituents that contribute to the complexity of the final wine^10,14,15^. Often giving a lower ethanol yield and being unable to complete fermentation^16^, they are used in sequential inoculation with *S. cerevisiae* to obtain wines with lower alcohol content, diverse aromas^17,18^ and flavour^6,19^. As such, they provide a solution to the increasing trend of high alcohol wines, which are in part attributable to climate warming^20–23^. Whilst several commercial starters containing different strains of *Torulaspora delbrueckii*, *Metschnikowia pulcherrima* and *Lachancea thermotolerans* are now available^24^, there is a multitude of species that remain to be investigated, including those specific to geographical wine regions that can be directly linked to the wines’ organoleptic characteristics.

The biodiversity of *Saccharomyces* strains is proposed to be as important as that of non-*Saccharomyces* in the sensorial qualities of the final wine^25^. Numerous reports exist that examine the biodiversity and distribution of individual strains isolated from vineyards and spontaneous fermentations from different wine regions in Europe^9,26–35^ as well as developing wine regions including China^36^, Brazil^37,38^, India^39^ and Russia^40^. Correlations have been made between the genetic diversity and agricultural practices^41^, vineyard diffusion of commercial starters^42,43^, grape variety^44,45^ and geographical distances^46^.

The recovery of minority or local grape varieties throughout Spanish regions has been undertaken to rescue genetic resources at risk of extinction^47–50^. These grape varieties can (i) lead to specific sensory characteristics in the corresponding wine, (ii) be better adapted to local climatic conditions, pests and diseases^51^ and may harbour novel yeasts. Negro Saurí (synonym Merenzao), an early maturing red variety^52^, is part of the Spanish Grapevine Breeding and Recovery Program by the Instituto Tecnológico Agrario de Castilla y León (ITACYL)^53^ which aims to produce new certified clones for the industry suited to the climatic and topographical conditions of the wine region. Whilst considered a minor variety, Negro Saurí is authorised in various appellations of origin such as Merenzao (e.g., Canary Islands, Galicia and Rioja) and has undergone resurgence within the León region because of its elegant aroma/flavour qualities as a varietal wine^54^. The contribution of associated microflora to these characteristics is unreported.

This study reports on the yeast microbiota and genetic relationships among *Saccharomyces* isolates from Negro Saurí grapes and spontaneously fermenting must from an experimental vineyard at Melgarajo S.A. (ITACYL) within the León Appellation of Origin, Spain. The purpose was to preserve the microbial genetic pool associated with this grape variety and establish a strain collection, which may be useful as starter cultures that help contribute to the regional character of the wines.

## Results

### Isolation and identification of yeast species from grape, must and spontaneous fermentation

We looked at the biodiversity of yeasts isolated from Negro Saurí grapes selected from an experimental vineyard (42° 15′ 48.68_N 5° 9′ 56.66_W). A total of 159 indigenous yeasts were isolated over four vintages: 2014 to 2017 from hand-harvested grapes, must and spontaneous wine fermentations (Table 1). The yeasts were identified through colony morphology and PCR analysis of the internal transcribed spacer 1 (ITS1), ITS4 and 5.8S ITS ribosomal DNA (rDNA) regions of the fungal genome^55^. Species were identified through comparison of the DNA sequence data from the different sized PCR bands to the available sequences in the NCBI database (GenBank) using the standard nucleotide homology search program Basic Local Alignment Search Tool^56^ (BLAST, http://www.nbci.nlm.nih.gov/BLAST).

**Table 1:** Diversity of yeast species isolated from different sources during four consecutive vintages (2014–2017).

Species	Source	Number of isolates			
		2014	2015	2016	2017
*Metschnikowia pulcherrima*	Grape	3	3	3	5
	Must	1	1	1	3
	Fermentation (start, mid, end)	(2, 1, 0)	(1, 0, 0)	(1, 0, 0)	(4, 3, 0)
*Lachancea thermotolerans*	Grape	2	–	–	–
	Must	1	–	–	–
	Fermentation (start, mid, end)	(1, 0, 0)	–	–	–
*Hanseniaspora uvarum*	Grape	-	–	–	–
	Must	1	–	–	1
	Fermentation (start, mid, end)	(2, 0, 0)	–	–	(4, 1, 0)
*Torulaspora delbrueckii*	Grape	–	–	–	-
	Must	–	–	–	-
	Fermentation (start, mid, end)	–	–	–	(0, 2, 0)
*Saccharomyces cerevisiae*	Grape	–	–	–	–
	Must	–	1	–	–
	Fermentation (start, mid, end)	(0, 5, 24)	(4, 5, 7)	(4, 5, 35)	(4, 6, 11)

Isolates identified by ITS PCR to species level, and by delta PCR for *Saccharomyces cerevisiae* strain differentiation.

**Figure 1:**
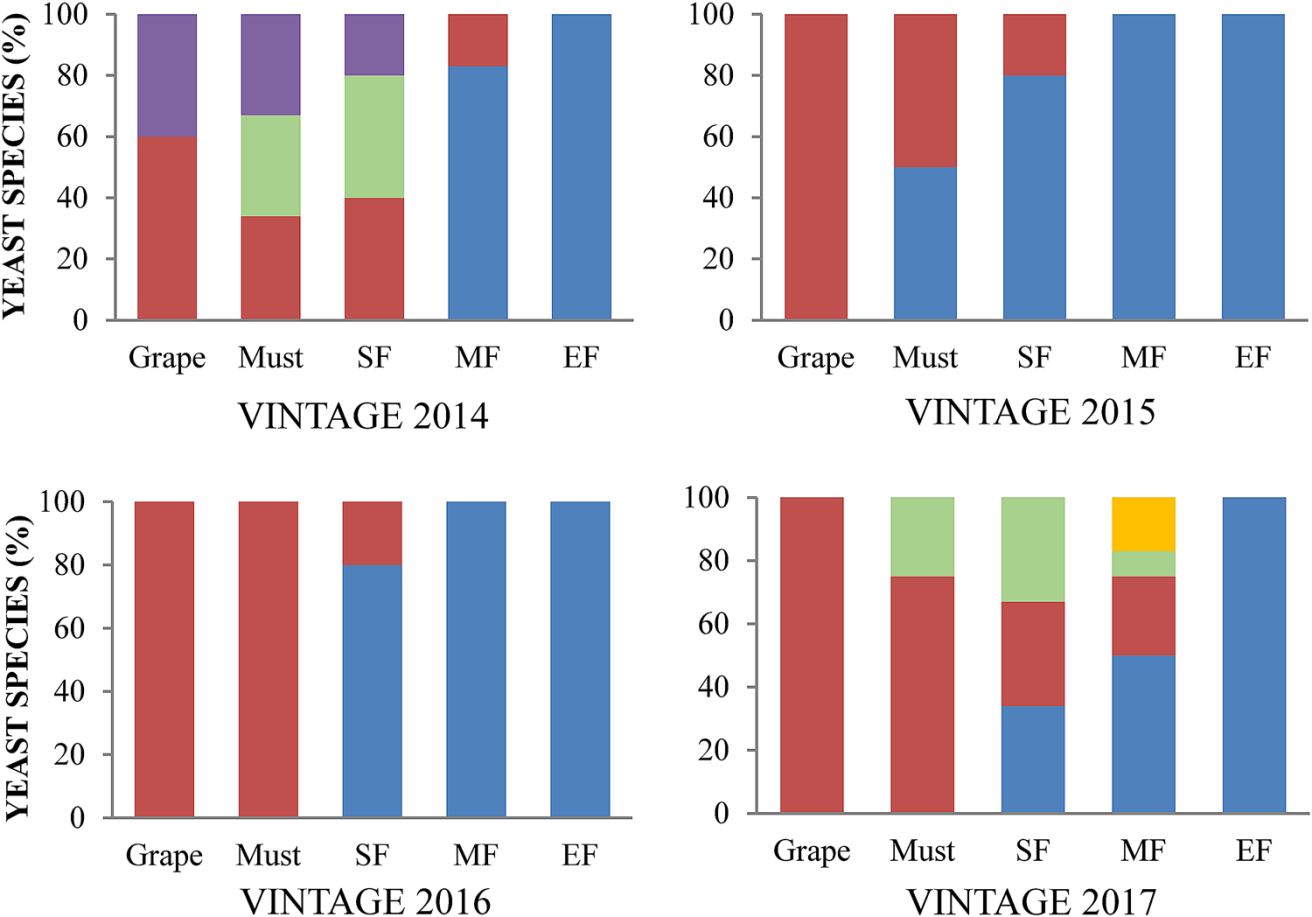
Biodiversity of yeast species on grapes, in must and during fermentation. SF, Start of fermentation; MD, Mid fermentation; EF, End of fermentation. *Saccharomyces cerevisiae* (blue), *Metschnikowia pulcherrima* (brown), *Hansenispora uvarum* (green), *Lachancea thermotolerans* (purple), *Torulaspora delbrueckii* (yellow). The species distribution was calculated dividing the number of isolates for a particular species by the total number of isolates.

*Saccharomyces cerevisiae* and 4 non-*Saccharomyces* species (*Metschnikowia pulcherrima*, *Lachancea thermotolerans*, *Hanseniaspora uvarum* and *Torulaspora delbrueckii*) were identified during the 4 consecutive vintages (2014–2017). The number of each species identified was dependent upon the source material (i.e., grape, must, spontaneous ferment) and unique vintage (Table 1). *Metschnikowia pulcherrima* was the sole species isolated from grapes in all vintages except for 2014, where *L. thermotolerans* was also identified (Fig. 1). The predominance of *M. pulcherrima* in grape must was less, with other species being present in near equal proportions, with the exception of *T. delbrueckii* and *S. cerevisiae*. *Hansenispora uvarum* was present during the 2014 and 2017 vintages, whilst *L. thermotolerans* was only found in 2014. *Saccharomyces cerevisiae* was noted in must in equal proportions to *M. pulcherrima* in 2015.

For each vintage, the spontaneous fermentations were sampled at the start, middle and end. Differences were noted between vintages in the species found (Fig. 1). Whilst *Saccharomyces* appear to outcompete the non-*Saccharomyces* species based on the number of isolates randomly picked and identified, differences were noted in how quickly this occurred (Table 1). In 2014, the yeasts identified at the start of fermentation were *M. pulcherrima* (40%)*, H. uvarum* (40%) and *L. thermotolerans* (20%). *Saccharomyces cerevisiae* did not dominate (83%) until mid-fermentation, whilst in 2015 and 2016, its more rapid dominance alludes to spontaneous fermentation being initiated prior to crushing (in 2015) or shortly afterwards (as in 2016). In both cases, *M. pulcherrima* was the only other species*.* In 2017, microbial succession was more complex, with the non-*Saccharomyces* species being prevalent during the start and mid-fermentation, with *S. cerevisiae* only dominating at the end fermentation.

Temperature and precipitation data (Supplementary Table S1 online) were collected to check for extreme seasonal variations that might have affected grape ripening, disease and quality; parameters which could influence microbial dynamics. Seasonal temperatures were similar in the 4 consecutive vintages. Rainfall varied—2016 had 29.4% more and 2017, 62.2% less than the average rainfall (413.5 mm; 2014–2017). Both years were considerably drier at harvest time (September)—2016 (49.1%) and 2017 (11.7%) of the average (22.4 mm). In 2014 and 2015, there was 72.3% and 66.9% more rainfall, respectively. The small sample size in this study (number of yeast isolates) especially from grapes and must, makes it difficult to make any comparisons between the individual microbial populations per vintage and the weather data.

### Genetic characterization of *Saccharomyces cerevisiae* strains

*Saccharomyces cerevisiae* represented the most abundant genus in the study. 110 indigenous isolates of *S. cerevisiae* were collected and analysed across four consecutive vintages (28 isolates (2014), 17 isolates (2015), 44 isolates (2016), 21 isolates (2017)). These were screened by inter-delta sequence analysis to evaluate genetic diversity and to determine their clonal relationships. Individual strains were identifiable through amplification of the delta sequences flanking the Ty1 retrotransposons within the yeast genome. The number and chromosomal position is strain-dependent^57^. Primers δ12 and δ21 were used since larger polymorphic differences can be identified, compared to the original δ1 and δ2 primers designed by Ness and co-workers^58^.

**Figure 2:**
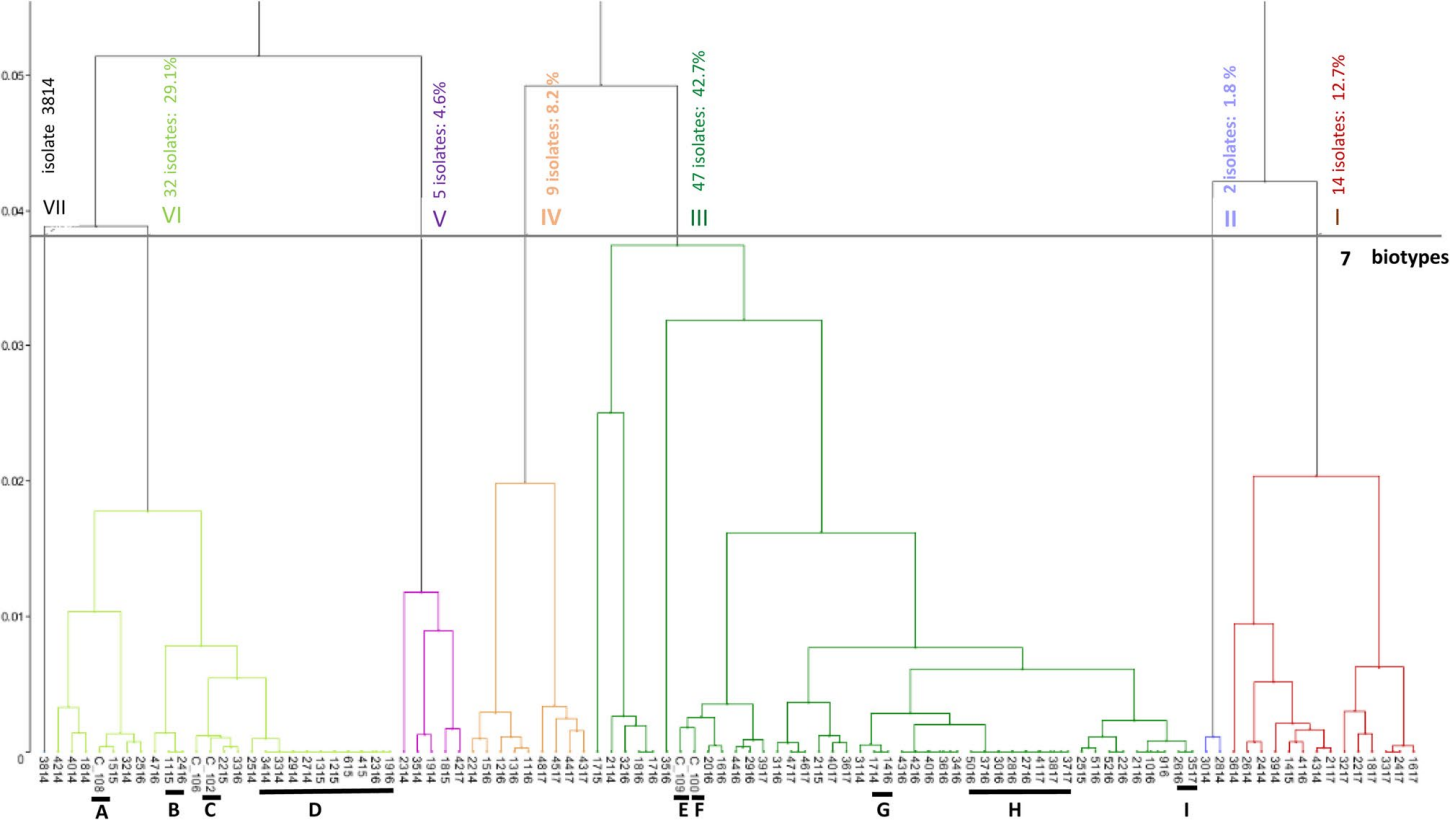
Global Ward dendrogram showing the genetic relationship of the 110 *Saccharomyces cerevisiae* isolates. Seven biotypes (I and VII) were identified based on the highest Calinski-Harabasz criterion value (18.717) and smallest Davies-Bouldin index value (1.052). In instances of > 100 isolates, the SPAD software names the most similar ones using a C number code (e.g. C_100). The isolates repeated among the vintages are as follows: C_100 (2415, 3816, 3916), C_102 (1514, 1614, 1915, 2015, 4516), C_108 (3714, 1615) and C_109 (2014, 2315). Another group, named C_106 includes the isolates 4616, 4816 and 4916 from the same vintage. The isolates for each C number are identical and represent individual strains (Supplementary Table S2 online). Letters A to I indicate nine perennial strains isolated from two or more vintages. CROSS was used as a reference strain, and forms part of Biotype I (Euclidean distance of 1.373) with the closest related strain, 3614 (from 2014) having a distance of 1.145 (Supplementary Table S2 online).

Typing analysis of the binary data from the amplified inter-delta sequence ‘fingerprint’ patterns was undertaken with Coheris Analytics SPAD software (2017), statistically analysed according to Lebart et al.^59^, and used the Euclidean distance and the Ward algorithm^60^. The number of partitions was based on the Davies-Bouldin and Calinski-Harabasz indicators^61^. To ensure accuracy, PCR analysis was repeated 4 times and in only one case was a strain discarded since results were not identical in at least 3 repetitions (data not shown). A total of 110 indigenous isolates were classified alongside a reference (Evo Cross (CROSS)) into seven clusters or biotypes, depending upon the presence or absence of 22 gel bands (Table 2). The genetic relationship of each of these groups is reported as a dendrogram (Fig. 2) with Multiple Correspondence Analysis (MCA) using the first factorial (F1) plane to display the (Euclidean) distances among clusters in the Ward’s dendrograms (data not shown). In order to describe each cluster quickly, value tests were calculated^59^ and the most significant modalities used. The distance of each individual isolate to the centroid of cluster allowing the identification of the homogeneity and similarity between these groups (data not shown).

**Table 2:** Delta PCR analysis of *Saccharomyces cerevisiae* strains.

Biotype (isolate count)	Band label	Category	% category in biotype	Biotype (isolate count)	Band label	Category	% category in biotype
VII (1)	50	0	100	III (47)	500	1	92.9
	1000	0	100		1200	0	92.9
	180	0	100		750	1	78.6
VI (32)	1200	1	94.4		1400	1	100
	450	1	94.4		150	1	100
	500	0	80.6		1300	1	23.8
	1400	1	100	II (2)	1000	0	100
	800	1	97.2		50	0	100
V (5)	300	1	60		800	0	100
	700	1	60	I (14)	1500	1	33.3
	150	0	60		750	0	86.7
IV (9)	550	1	66.7		450	0	73.3
	1100	1	44.4		1200	0	93.3
	1200	0	100		800	0	40
	450	0	77.8		1400	0	86.7
					50	0	100

Twenty-two PCR amplicon sizes were scored as binary data (0: absence, 1: presence of band) and used for dendrogram construction depicting the genetic relationships between the various strains. Test value: p < 0.05. Seven biotypes were identified (I to VII), with the number of isolates per biotype shown in brackets.

### Global dendrogram identifying strain diversity over four consecutive vintages

The study of 110 *S. cerevisiae* isolates from the four single spontaneous Negro Saurí fermentations undertaken identified not only strains specific to a single vintage, but those recoverable in successive vintages as well as clonal isolation of individual strains (Fig. 2, Table 3, and Supplementary Table S2 online). The 110 isolates represent 70 genetically distinct strains collected over the four vintages during different stages of fermentation. The percentage of genetically distinct strains amongst the total collected varied per vintage (85.7% (2014), 76.5% (2015), 63.6% (2016) and 80.9% (2017)). Seven major biotypes (Biotypes I–VII) were identified amongst the *S. cerevisiae* isolates (Fig. 2, Tables 2 and 3). 2014 showed the greatest variability with 25 strains identified amongst the 28 isolates, which were distributed between the 7 biotypes (I (41.6%), II (100%), III (6.4%), IV (11.1%), V (60%), VI (37.5%) and VII (100%); Fig. 2). 2016 exhibited the smallest genetic variability despite the number of isolates sampled with a high degree of clonality observed for this vintage, which is discussed below. The following biotypes lacked isolates from vintages 2015 (IV), 2016 (V) and 2017 (VI).

**Table 3:** Distribution of seven *Saccharomyces cerevisiae* biotypes found in Negro Saurí must and during spontaneous fermentation.

Biotype	I	II	III	IV	V	VI	VII	Σ
	**2014**							
M^a^								28
SF								
MF			1714	2214	1914	1514 1614		
EF	2414 2614 3614 3914 4314	2814 3014	2014 2114 3114		2314 3514	1814 2514 2714 2914 3214 3314 3414 3714 4014 4214	3814	
	**2015**							
M						0415		17
SF	1415					0615 1115 1215 1315		
MF			1715		1815	1515 1615 1915		
EF			2115 2315 2415 2515			2015 2215		
	**2016**							
M^a^								
SF			0916 1016	1116 1216				44
MF			1416 1616 1716	1316 1516				
EF	4116		1816 2016 2116 2216 2616 2716 2816 2916 3016 3116 3216 3416 3516 3616 3716 3816 3916 4016 4216 4316 4416 5016 5116 5216			1916 2316 2416 2516 3316 4516 4616 4716 4816 4916		
	**2017**							
M^a^								21
SF	1617 1817 2117 2217							
MF	2417 3217 3317		3517 3617 3717					
EF			3817 3917 4017 4117 4617 4717	4317 4417 4517 4817	4217			
Reference	CROSS							1
Σ	16	2	47	9	5	32	1	111

*Saccharomyces cerevisiae* isolates were identified using a 4-numeral code; first two numbers (isolate) and the last two numbers (vintage). Biotypes refer to the Global Ward dendogram (Fig. 2). M, must; SF, start of fermentation; MF, mid-fermentation; EF, end of fermentation; CROSS, Cross Evolution; ^a^, not available for must (2014, 2016, 2017).

Biotypes II and VII were considered as “vintage medium” biotypes, defined by bands that did not identify the rest of the biotypes. In this case, the absence of bands 50, 1000, and 180 for Biotype VII and 50, 800 and 1000 for II, respectively (Table 2). These 2 biotypes had genetically distinct strains specific to 2014 (II; 2814, 3014 and VII; 3814). Biotype III (representing 42.7% of total) was largely represented by isolates from 2016 of which there was a high number of clonal isolates. This probably reflects the larger sample size (47) compared to the other vintages (Table 3). Three strains were specific to this vintage: 1 strain was present at the beginning and end of fermentation (SF: 0916, 1016; EF: 2016), whilst another 2 strains were confined to the EF ((2416, 3616, 4016, 4216, 4316) and (2216, 5216)). Biotype IV (representing 8.2% of total isolates) had 9 genetically similar but distinct strains from the first (2214), third (1116, 1216, 1316, 1516) and fourth vintage (4317, 4417, 4517, 4817). Whilst Biotype V (4.6% of total) consisted of 5 distinct strains; 1914 and 1815 isolated from mid-fermentation whilst 2314, 3514 and 4217 were found at the end of ferment (Table 3). In Biotype I (12.7% of total), 13 of the 14 isolates represented different strains, with only 2 isolates being clonal isolates (3317, 2417), which were from mid-ferment. The temporal distribution of these strains indicates a ‘flow’ of individual strains during the course of fermentation. For example, during vintage 2014, four strains belonging to Biotype I were found at EF (2414, 2614, 3614, 4314), whilst 1415 was at SF in 2015, and 4116 was at the EF (vintage 2016). In 2017, 4 strains (1617, 1817, 2117 and 2217) were from the start of fermentation, whilst 3 were from mid-fermentation (2417, 3217 and 3317).

Clonal enrichment of strains was observed for 3 biotypes (VI, III and I) and was typically observed at the end of fermentation (Fig. 2). The number of clonal isolates varied between 2 and 4 or 5, depending on the biotype and vintage (Table 3). Some strains were identified within a single vintage and are ‘annual’ strains, such as 4 strains belonging to Biotype III isolated only in 2016 (1816, 1716), (5216, 2216), (916, 1016, 2116) and (3416, 3616, 4016, 4216, 4316). Another 2 strains (Biotype III: 4717, 4617) and (Biotype I: 2417, 3317) were only observed in 2017. Other strains were ‘perennial’, being present in at least two vintages though not necessarily consecutive ones. For example, within biotype III, there were four different strains E (C_109: 2014, 2315, 3917), F (C_100: 2415, 3816, 3916), G (1714 and 1416), and H (2716, 2816, 3016, 3716, 5016, 3717, 3817, 4117) (Supplementary Table S3 online).

More importantly, in terms of useful starter cultures, a total of 9 ethanol tolerant strains (A to I) were found to dominate in two or more vintages (Fig. 2). For example, one strain (D) was identified amongst four isolates in Biotype VI (2714, 2914, 3314, 3414) at the end of fermentation in 2014, to be reisolated in 2015 in the must (M: 415) and at the start of fermentation (SF: 615, 1215, 1315). Strain D reoccurred again in 2016 at the end of fermentation (EF: 1916, 2316). Another strain (H) identified as clonal isolates in Biotype III in 2016 (EF: 2716, 2816, 3016, 3716) was similarly observed the following year (MF: 3717, EF: 3817, 4117). These findings suggest that these particular strains are endemic to the winery but do not identify as Evo Cross (CROSS), which is used as the reference strain and is part of Biotype I, with the closest related strain being 3614 (Fig. 2).

In conclusion, the *S. cerevisiae* population in Negro Saurí spontaneous fermentations appears to be both diverse and transient, with only a few strains being prominent over several vintages. Seventy distinct strains were identified over the four vintages, whilst the sum of strains per vintage was 82 (24 strains (2014), 13 strains (2015), 28 strains (2016), and 17 strains (2017); Fig. 2) as 12 strains were present in one or more vintages. Of the 9 ‘winery related’ strains, 7 were found across 2 vintages (A, B, E, F, G, H, I) and only 2 (C, D) persisted over 3 consecutive years. None were found to persist over the 4 years. Whilst the study revealed 61 ‘single vintage’ strains, which may be worthy of further investigation in terms of their oenological properties. More interesting are the 9 strains that were more persistent, as shown by their being identified in 2 or more vintages. Larger scale fermentations using these strains as starter cultures are required with an in-depth sensory and chemical analysis to determine whether they are useful to the local wine industry in terms of providing an aroma profile typical of this locality’s ‘microbial terroir’.

## Discussion

We report for the first time on the selection and genetic characterization of autochthonous microorganisms from the minority variety, Negro Saurí. The goal was to preserve the genetic resources of the locality, whilst providing insights into the wine strain diversity and, in turn, their role in contributing to the aroma-flavour profile of regional wines. This study aligns with the increasing demand for indigenous *S. cerevisiae* isolates that are representative of a specific oenological area^62^, with particular wineries increasingly selecting yeasts within their vineyard for specific characteristics and suitability to local grape varieties^63,64^. Extensive studies of spontaneous fermentations in Italy^65^, Spain^18,66^, USA^67^ and Canada^68^ (including studies specifically of the genetic diversity of *Saccharomyces cerevisiae* in different wine-producing regions^36,69,70^) are validated or partly motivated by reports^71^ of a significant correlation between the region from where the strains were isolated and the aroma profile of the resulting wines.

In this study, four non-*Saccharomyces* yeasts (*Metschnikowia pulcherrima, Lachancea thermotolerans, Hanseniaspora uvarum and Torulaspora delbrueckii*) were isolated from Negro Saurí grapes and must, based on their colony morphology and subsequent ITS sequencing. This result is similar to earlier reports of the microbiota associated with Prieto Picudo, grown in the León Appellation of Origin^72^, and Carinyena and Garnacha grapes from the Priorat wine region of Tarragona, Spain^73^. The lack of diversity seen in all three studies most likely relates to the isolation procedure and the restricted number of isolates tested. Plating of dilutions of grape, must and ferment samples onto nutrient-rich media favouring the more numerous, and faster-growing species over minor, slower growing ones^74^. No interpretation could be made from the small numbers isolated in relation to grape variety, geographic location and climatic conditions and yeast diversity and number^75^. However, the reduced number of culturable species may relate to the use of organic (sulphur, copper) and inorganic fungicides (Supplementary Table S3 online), corroborating published data on the influence of fungicide spray regimes on yeast diversity and number^76–79^. Whilst these fungicides target *Odium* and other mildew fungi, they also inhibit other ascomycetes including wine non-*Saccharomyces* because of their broad mode of action. For example, ergosterol biosynthesis (triazoles—penconazole, cyproconazole, propiconazole), RNA synthesis (phenylamides—metalaxyl), mitochondrial respiration (synthetic strobilurins—trifloxystrobin), and arylaminopyridines (fluazinam), whilst others are non-specific (phthalimides—captan, folpet)^80^.

*Aureobasidium pullulans* is a well-documented species on grapes; its presence being independent of variety or viticultural practise^77^. The use of elemental sulphur and copper (as oxychloride and cuprocalcium sulphate) has resulted in its adaptation, with the fungus able to detoxify sulphur^81^ and copper^82^. In this study, fungal colonies were isolated, but not confirmed as *Aureobasidium pullulans* because of their low relative abundance compared to *Metschnikowia *sp., *L. thermotolerans*, *H. uvarum* and their inability to ferment, making them of limited oenological interest^83,84^. *Metschnikowia pulcherrima* was successively isolated from grapes and must and to a lesser extent, in the early stages of fermentation. *L. thermotolerans* and *H. uvarum* featured in 2014 and 2017, on grapes, in must and during early fermentation. In 2017, species diversity included *T. delbrueckii*, which was isolated from mid-fermentation samples. *Metschnikowia spp* have also been associated with Prieto Picudo, which is the main grape variety in the León Appellation of Origin^72^. Whilst the authors did not report on *L. thermotolerans,* this species is both genetically (and phenotypically) diverse^85^, and has a diverse geographical distribution^86^. *Hanseniospora uvarum* is well known to be abundant on grapes^74^ and can even predominate at the start of an un-inoculated (spontaneous) fermentation^16,65^ and in some instances in the final stages^87^. In our study, *Hanseniospora* could not be isolated from the aseptically sampled grape berries, but were present in the grape must and fermentation samples taken at the experimental winery. These findings, similar to those of Grangeteau and coworkers in France^15^, allude to implantation of the must from winery environment, with the possibility of some non-*Saccharomyces* species persisting from one year to another in the winery environment to later dominant during fermentation.

The non-*Saccharomyces* yeasts identified in our study and by others are of potential oenological interest. For instance *Metschnikowia pulcherrima* offers possibilities in biocontrol of spoilage yeasts^88^ whilst the use of *M*. *pulcherrima*, *L. thermolerans* and *T. delbrueckii* offer paradigms for reducing wine ethanol content^89^, in deacidification and improving aroma complexity^90,91^. Furthermore, the production of extracellular enzymes such as pectinases can aid anthocyanin extraction, clarification and filterability^92^. Likewise, *H. uvarum* can secrete β-glucosidases, which can release glycosidically bound grape-derived terpenes, thereby contributing to varietal aroma in wines^14,93^. ‘Tailored’ autochthonous starter cultures are of interest not only in terms of ‘terroir’ but provide safer alternatives to spontaneous fermentations in relation to human health, as microbial producers of contaminants (e.g., biogenic amines, ethyl carbamate, etc.) are screened for (reviewed in^94^). To date, there are only a small number of commercial starter cultures available for co- or mixed culture fermentations^95^. As such, there is a continued need for specific strains, with distinctive properties in terms of novel wine styles, and improving wine quality and process efficiency.

Whilst non-*Saccharomyces* yeasts are easily isolated from the grape surface^16^, the extremely low occurrence of *S. cerevisiae* on healthy, undamaged grapes, makes it largely undetectable^96^, and difficult to recover without enrichment through a fermentation^97^. In this study, spontaneous fermentation was conducted each vintage in sanitised 500 L steel tanks at the experimental winery. *S. cerevisiae* was typically isolated at the start of fermentation with the exception of 2014 when it was not evident until mid-fermentation, and 2015 when it was largely present in the grape must, alluding to possible fruit damage prior to harvesting, so promoting *S. cerevisiae* growth. In all cases*, S cerevisiae* typically dominated and completed the fermentation.

The 110 *S. cerevisiae* isolates were classified by their delta sequence patterns. Heterogenous populations were found both annually and over the four-year study period. In total, 70 strains were identified, of which the majority (61) were confined to a single vintage and most likely represent the indigenous population in the vineyard, which was dynamic and vintage dependent. Nine strains (Fig. 2A–I) were isolated in 2 or more consecutive vintages suggesting that they are part of the resident microbiota of the winery at Melgarajo S.A., which was 3 km from the vineyard and 17 km from the nearest neighbouring winery. Similar findings are reported by Clavijo et al.^98^, although the authors identified commercial strains in the laboratory fermentations, and concluded that the strains were transferred from the winery into nearby vineyards through insect transmission, the spreading of yeast lees in the vineyard as well water run-off from routine cellar operations. The yeasts persisted in only a single vintage, which is contrary to our ‘winery implanted’ strains. Whilst insect transfer from the winery or between neighbouring plots is possible, the strains are likely to be indigenous given that commercial strains were not used for experimental winemaking. There is still some debate as to whether grape variety or location may influence the indigenous *Saccharomyces* population. Clavijo et al.^98^ reported on separate populations in 3 grape varieties (Syrah, Merlot and Cabernet Sauvignon) whilst Capece’s research^99^ examining 11 varieties grown in a single vineyard, alluded to specific native *S. cerevisiae* strains being associated with a particular terroir. However, Santamaría and co-workers^100^ in a recent study of 11 wineries in the Rioja region showed that the strains varied with vintage and none were common to neighbouring wineries or the area. In a much larger study using delta PCR sequencing, Sun et al.^36^ concluded that not only do *Saccharomyces* populations differ between grape varieties, but also between wine and table grapes, as well as geographical location both on a local (region) and global (country) scale.

This is the first report of the microbiota associated with the minor red variety Negro Saurí. The objective of the study was to capture and preserve the yeast diversity, with a view to such strains being potentially used as starter cultures and/or contributing to a distinct terroir. The nine identified perennial *Saccharomyces* isolates require further characterisation to determine their suitability, a task beyond the scope of this initial ‘identify and survey’ study.

## Material and methods

### Experimental vineyard and management

The study was based at an experimental vineyard at Melgarajo S.A. (42° 15′ 48.68_N 5° 9′ 56.66_W), which is located in Melgar de Abajo within the León Appellation of Origin. Three rows of Negro Saurí grapes (15 cultivars) were cultivated as part of the ITACYL program to rescue this variety (Supplementary Fig. S1 online). The grapes used in the study were from these cultivars.

Viticultural management (fungicide, herbicide, vine nutrition) was recorded for each growing season (Supplementary Table S3 online). Glyphosate was used as a post-emergence herbicide each season. Sulfur (98%) was used as the organic fungicide and acricide in 2014 and 2017, whilst Caldo Bordeles (cuprocalcium sulphate) was used in 2015, and a combination of sulphur and Corbre Lainco (copper oxychloride) in 2016. Other organic sulphur fungicides used included dithiocarbamates such as mancazeb (Fantic-M). Inorganic fungicides were rotated annually, with several classes (differing in mode of action) used singularly and in combination to prevent fungal resistance (Supplementary Table S3 online).

The experimental facility (winery) used in the study was 3 km from the vineyard Winery equipment purchased for sole use with un-inoculated fermentations, with no commercial *Saccharomyces* used on the premises. The nearest winery was 17 km from Melgarajo S.A.

#### Sampling procedure

Samples were collected at maturity (~ 24°Brix) over 4 consecutive vintages: 2014, 2015, 2016 and 2017. Grape samples (1 kg) were aseptically collected, placed into sterile plastic bags and transported to the Microbiology Laboratory at the Veterinary Faculty of the University of León for analysis. Additional grapes (~ 500 kg) were hand-picked into 20 kg boxes, before being destemmed, pressed and the must transferred to potassium metabisulfite-treated steel tanks (500 L) to undergo un-inoculated (spontaneous) fermentation. Fermentation was conducted at 16–18 °C at the experimental facility within Melgarajo S.A., which is used only for research wine production of Negro Saurí. Wine samples (100 mL) were taken twice-weekly during the fermentation. For chemical data for Negro Saurí grapes and wine refer to Supplementary Table S4 online.

#### Yeast isolation from grapes and spontaneous fermentation

The 1 kg grape samples were aseptically homogenized using a Stomacher 400 laboratory paddle blender (Seward Ltd., England) for 1 min at normal intensity. Aliquots (0.1 mL) of homogenized grapes and wine samples were plated at different serial dilutions using 0.9% saline onto YED agar (1% yeast extract, 2% glucose and 2% agar; pH 4.5) supplemented with 150 µg mL^−1^ chloramphenicol (Sigma-Aldrich, St. Louis, Mo, USA) to inhibit bacterial growth. The plates were incubated at 30 °C for 2–3 days after which, the various colony types were isolated at random according to shape, colour, surface feature and frequency^101^. The yeast cultures were preserved at − 80 °C in liquid YED medium with glycerol 30% as a cryoprotectant ahead of identification.

#### S rRNA gene analysis

Genomic DNA was extracted according to Lõoke et al.^102^ using either single yeast colonies (or cells collected from 100 μL of liquid YPD culture (OD_600_ 0.4)). The DNA pellet was dissolved in 100 μL water and the debris removed by centrifugation (15,000×*g* for 1 min) before one μL of supernatant was used for PCR. The 5.8S rRNA gene analysis was undertaken according to Esteve-Zarzoso et al.^55^, with the internal transcribed spacer regions (ITS1 and ITS4) used for yeast identification. PCR amplification was performed in a 50 µl reaction with 100 ng genomic DNA as template, 0.1 µM each primer, 0.4 mM dNTP and 0.5 unit of BiTOOLS DNA polymerase (ULTRATOOLS, Spain). The primers used for amplification were ITS1 (5′-TCCGTAGGTGAACCTGCGG-3′) and ITS4 (5′-TCCTCCGCTTATTGATATGC-3′). PCR amplification was initiated with a 5 min denaturation at 95 °C, followed by 35 amplification cycles (94 °C, 1 min; 55.5 °C, 2 min; 72 °C, 2 min) and terminated with a 10 min extension at 72 °C. PCR products were separated on 0.5 and 3% agarose gels in 1× TAE buffer (40 mM Tris, 20 mM acetic acid, 1 mM EDTA (pH 8). Products were purified using Wizard SV Gel and PCR Clean-Up (Promega) and sent to the DNA Sequencing Service of the Laboratory of Instrumental Techniques of the University of León. Sequence comparisons were performed using the basic local alignment search tool (BLAST) program within the NCBI database^56^.

#### Genetic characterization of *Saccharomyces cerevisiae* strains using delta PCR analysis

The genetic diversity within 111 strains isolated from spontaneously fermented Negro Saurí juice and one commercial wine *Saccharomyces cerevisiae* (Cross Evolution, Lallemand) was evaluated by PCR amplification of inter-delta (δ) regions, which flank Ty elements in the yeast genome^58^. DNA isolation was performed according to Liu et al.^103^. PCR amplification of inter-delta sequences was with primers δ12 (5′-TCAACAATGGAATCCCAAC-3′) and δ21 (5′-CATCTTAACACCGTATATGA-3′)^57^. PCR amplification was performed in a 25 µl reaction using 30–100 ng template DNA, 0.8 µM each primer, 0.2 mM dNTP and 2.5 unit of Mango Taq (Bioline). PCR conditions were as follows: initial denaturation for 4 min at 95 °C followed by 35 cycles of 30 s at 95 °C; 30 s at 46 °C and 90 s at 72 °C, and a final extension step of 10 min at 72 °C. The PCR products were separated on 2% agarose gels and the size of the amplified DNA fragments estimated with 50 bp HyperLadder DNA markers (Bioline).

PCR amplification was repeated four times per isolate to ensure repeatability, with the results considered accurate when 3 or 4 replicates were equal; where this did not occur, the results were reviewed.

### Statistical analysis

The interdelta sequence patterns were used to construct a presence/absence matrix (binary 0/1^69^). All visible bands were assigned a number based upon relative position to the DNA ladder, with each position assigned a score to indicate the presence (1) or absence (0) of the band (Table 2).

The 110 isolates and Cross Evolution (referred to as CROSS) were classified on the basis of similarity among patterns of the 22 bands, depicted in a global dendrogram. For dendrogram construction, the statistical analysis was carried out according to Lebart et al.^59^. Firstly, due to the data described being 0 or 1, the strains were analysed by applying Multiple Correspondence Analysis (MCA^104^). For the construction of the global dendrogram, a hierarchical cluster analysis (Euclidean distance and Ward algorithm^60^) was applied, taking the components retained (Kaiser's Criterion) from the MCA analysis. Finally, the best partition of the dendrogram was based on the Davies-Bouldin and Calinski-Harabasz indicators^61^. The statistical software Coheris Analytics SPAD (2017) version 9.0.39 was used.

## Supplementary Information

Supplementary Information.
