# Author Correction: Capturing yeast associated with grapes and spontaneous fermentations of the Negro Saurí minority variety from an experimental vineyard near León

**DOI:** 10.1038/s41598-021-89721-3

**Published:** 2021-05-04

**Authors:** Isora González-Alonso, Michelle Elisabeth Walker, María-Eva Vallejo-Pascual, Gérmán Naharro-Carrasco, Vladimir Jiranek

**Affiliations:** 1 Department of Animal Health, Universidad de León, Campus de Vegazana, León, Spain; 2 Department of Wine Science, The University of Adelaide, Waite Campus, Urrbrae, SA 5064 Australia; 3 Department of Economy and Statistics, Universidad de León, Campus de Vegazana, León, Spain; 4 Australian Research Council Training Centre for Innovative Wine Production, Adelaide, Australia

Correction to: *Scientific Reports* 10.1038/s41598-021-83123-1, published online 12 February 2021

The original version of this Article contained a typographical error in the spelling of the author María-Eva Vallejo-Pascual, which was incorrectly given as María-Eva Pascual-Vallejo. This has now been corrected in the PDF and HTML versions of the Article, and in the accompanying Supplementary Information file.

