# Supplementary material for: Capturing yeast associated with grapes and spontaneous fermentations of the Negro Saurí minority variety from an experimental vineyard near León
Source: Sci Rep. 2021 Feb 12;11:3748. doi: 10.1038/s41598-021-83123-1 (PMC7881026; doi:10.1038/s41598-021-83123-1)
Supplement: Supplementary file 1 — Supplementary Information. [file 41598_2021_83123_MOESM1_ESM.pdf]

Capturing yeast associated with grapes and spontaneous fermentations of the Negro Saurí minority variety from an experimental vineyard near León

Isora González-Alonso[1], Michelle Elisabeth Walker[2], María-Eva Vallejo-Pascual[3], Gérmán Naharro-Carrasco[1], and Vladimir Jiranek[2,4*]

[1] Department of Animal Health, Universidad de León, Campus de Vegazana, León, Spain
[2] Department of Wine Science, University of Adelaide, Waite Campus, South Australia, Australia
[3] Department of Economy and Statistics, Universidad de León, Campus de Vegazana, León, Spain
[4] Australian Research Council Training Centre for Innovative Wine Production, Adelaide, Australia

Supplementary Fig. S1. Map of experimental vineyard at at Melgarajo S.A.

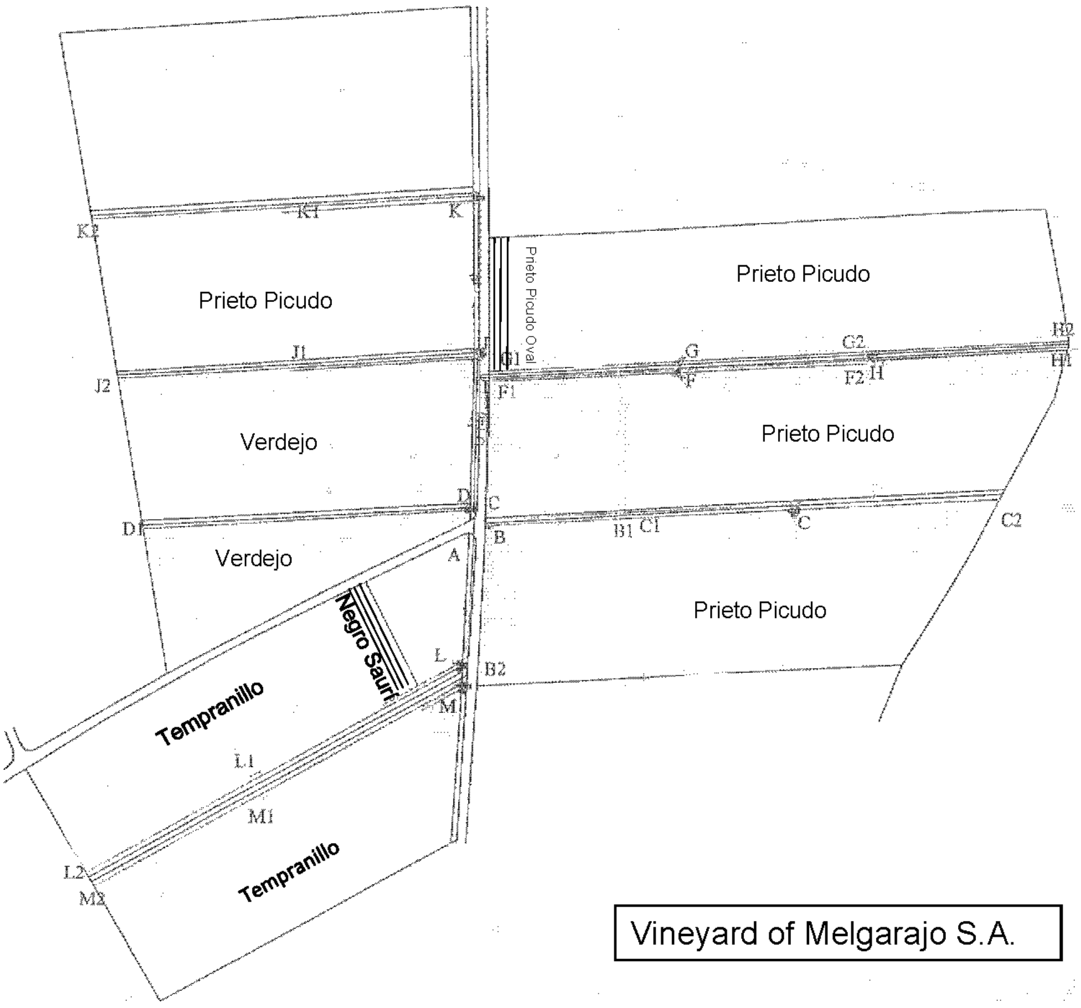

L1 Negro Sauri (3 rows, 15 cultivars; 300 plants (used in the study). Tempranillo grapes are sold.

M1 Tempranillo (grapes are sold)

D and E Verdejo (commercial wine is made off-site and not in experimental winery)

B, F, G and K Prieto picudo (commercial wine is made off-site and not in experimental winery)

G Prieto Picudo oval (clonal research).

An empty plot (behind K) to be planted with clones (from ITACYL's Negro Saurí Research) for future wine production

Supplementary Table S1. Average temperature (Tm; ºC) and precipitation (P; mm) for the period 2014-2017 (October 2013 - September 2017) as registered at the meteorological station of Mayorga de Campos, Valladolid.

	Oct	Nov	Dec	Jan	Feb	Mar	Apr	May	Jun	Jul	Aug	Sep	Ave
**Tm 2014**	12.4	5.8	2.0	5.5	5.0	7.8	12.3	13.5	18.1	19.8	19.7	18.1	11.7
**Tm 2015**	14.6	8.5	3.4	1.6	3.7	8.1	10.9	14.7	19.4	22.7	20.2	15.4	12.0
**Tm 2016**	11.8	8.0	4.9	5.4	5.2	5.9	8.4	12.3	18.3	22.3	21.6	17.7	11.8
**Tm 2017**	12.8	6.5	3.8	2.3	6.3	8.2	11.4	15.7	21.3	21.6	20.8	16.2	12.3
	**Oct**	**Nov**	**Dec**	**Jan**	**Feb**	**Mar**	**Apr**	**May**	**Jun**	**Jul**	**Aug**	**Sep**	**Total**
**P 2014**	82.2	11.5	60.0	58.6	63.6	21.8	33.3	22.4	10.3	26.8	1.2	38.6	430
**P 2015**	4.0	89.6	26.3	33.7	14.5	9.0	53.8	23.1	121.2	17.1	2.8	37.4	432
**P 2016**	84.2	42.2	22	105.2	47.4	31.6	106.4	78.4	2	1.2	3.6	11	535
**P 2017**	29.2	22.8	21	20.4	56.4	13.6	7.87	30.9	22.21	14.54	15.15	2.63	257

Supplementary Table S2. Global MCA: eigenvalues, cluster selection, biotypes, strains and distances

GLOBAL MCA: 110 strains and CROSS strain (supplementary) – 22 gel bands

TABLE 1: EIGENVALUES AND RETAINED FACTORS FOR CLUSTERING
9 factors - 64,5 %

Factor	Variance of the Factor (eigenvalue)	% of explained variance	Cumulated % of explained variance	Benzécri's modified rates (%)	Retained according to Kaiser's criterion
**1**	0,110	11,0	11,0	40,5	X
**2**	0,106	10,6	21,5	35,4	X
**3**	0,076	7,6	29,1	9,0	X
**4**	0,071	7,1	36,2	6,3	X
**5**	0,068	6,8	43,0	4,8	X
**6**	0,061	6,1	49,1	2,5	X
**7**	0,056	5,6	54,7	1,0	X
**8**	0,053	5,3	59,9	0,5	X
**9**	0,046	4,6	64,5	0,0	X

TABLE 2: CLUSTER SELECTION
highest Calinski-Harabasz criterion; smallest Davies-Bouldin's index

Criteria	5 clusters	6 clusters	**7 clusters**
Intra-cluster inertia	0,392	0,350	**0,309**
Inter-cluster inertia	0,254	0,295	**0,337**
Explained inertia (%)	39,302	45,751	**52,160**
Calinski-Harabasz (pseudo F) criterion	16,997	17,542	**18,717**
Davies-Bouldin's index	1,385	1,203	**1,052**

TABLE 3: 7 biotypes, strains and distances

I

Strain	Distance
1415	0,277
4116	0,299
2117	0,34
4314	0,392
2217	0,465
3914	0,5
1817	0,617
1617	0,623
2417	0,687
3317	0,687
2414	0,712
3217	0,75
2614	0,813
**3614**	**1,145**
**CROSS**	**1,373**

II

Strain	Distance
2814	0,192
3014	0,192

III

Strain	Distance
3416	0,234
3616	0,234
4016	0,234
4216	0,234
4316	0,234
916	0,263
1016	0,263
2116	0,263
3717	0,280
3817	0,280
4117	0,280
2716	0,280
2816	0,280
3016	0,280
3716	0,280
5016	0,280
2515	0,289
2216	0,292
5216	0,292
3114	0,319
2916	0,340
2616	0,353
3517	0,353
4416	0,368

III continuation

Strain	Distance	Dendrogram code
1416	0,392	
1714	0,392	
2315	0,410	C_109
2014	0,410	
3917	0,414	
5116	0,429	
3116	0,432	
3816	0,458	
3916	0,458	C_100
2415	0,458	
4617	0,494	
4717	0,494	
2016	0,522	
3617	0,553	
4017	0,575	
2115	0,577	
1616	0,633	
3216	0,993	
1716	0,998	
1816	0,998	
2114	1,055	
1715	1,635	
3516	1,863	

IV

Strain	Distance
4317	0,774
4417	0,574
4517	0,778
4817	0,702
1116	0,537
1216	0,511
1316	0,653
1516	0,587
2214	0,472

V

Strain	Distance
1815	0,492
3514	0,610
4217	0,668
1914	0,714
2314	1,018

VI

Strain	Distance	Dendrogram code
4516	0,148	
1915	0,148	
2015	0,148	C_102
1514	0,148	
1614	0,148	
1916	0,241	
2316	0,241	
415	0,241	
615	0,241	
1215	0,241	
1315	0,241	
2714	0,241	
2914	0,241	
3314	0,241	
3414	0,241	
2215	0,288	
2516	0,299	
3316	0,310	
4616	0,333	
4816	0,333	C_106
4916	0,333	
2514	0,454	
1615	0,478	C_108
3714	0,478	

VI continuation

Strain	Distance
3214	0,513
1515	0,519
2416	0,595
1115	0,595
4716	0,607
1814	0,765
4214	0,782
4014	1,031

VII

Strain
3814

Supplementary Table S3. Phytosanitary products used at Melgarajo

**2014**<br>**Agent**	 **Description**
**TOPAS (TOPAS 100EC)**	penconzaole 10%; control of powdery mildew
**THIOVIT JET**	sulfur 80%; control of powdery mildew, rust, mites
**CHOKE**	
**PROFOL PLUS 6-30-37**	
**AZUFRE ESPOLVOREO OROFLEX**	sprayable sulfur; broad spectrum fungicide
**ASSISTAN**	pre- and post-emergence herbicide
**ATILA**	glyphosate 36%; post-emergence herbicide
**LATINO**	miclobutanil 12.5%; control of *Odium* spp (powdery/downy mildew)
**CYCLO PLUS**	folpet 40% + metalaxyl 10%; control black rot, mildew
**APACHE**	chlorothalonil 37.5% + propiconazole 6.25% + cyproconazole 5%; broad spectrum fungicide
**SFENVALO STAR**	esfenvalerate 2.5%; insecticide
**NUTRACEICO**	
**EMERALD**	Fluazinam 50%; fungicide
**AMICOS COMBI**	Plant extract; anti fungal sporulant, micronutrient supply (Mn, Zn)
**SUFREVIT**	sulfur 80%; fungicide, acaricide
**CERCOBIN**	broadspectrum fungicide; polyvalent; systemic and contact.
**2015**<br>**Agent**	 **Description**
**ATILA**	glyphosate 36%; post-emergence herbicide
**PROWL**	pendimethalin 33%; herbicide
**MEDELINON FLOW**	linuron 45%; systemic herbicide
**FLINT**	trifloxistrobin 50%; control of *Odium* spp
**ORTHOCIDE**	captan 80%; control of mildew
**DEEPEST TIRAM**	tiram 50%; broad spectrum fungicide
**BRANDA METALAXIL**	metalaxyl; control of mildew
**LAITRI**	miclobutanil 12.5%; control of *Odium* spp
**FANTIC-M**	benalaxil M 4% + mancozeb 65%; control of mildew
**CALDO BORDELES LAINCO**	copper 20% (cuprocalcium sulfate); broad spectrum fungicide
**2016**<br>**Agent**	 **Description**
**TACTIC**	organosilicone surfactant
**BORIGAN**	wetting agent
**GRANO DE ORO (Azufre)**	98.5% sulfur; fungicide
**COBRE LAINCO**	copper oxychloride 50%; control of mildew

**LAINCOIL ACEITE VERANO**	summer oil 83%; pesticide
**KARDA GLIFOSATO**	glyphosate; post-emergence herbicide
**PRINALUM**	pendimethalin 33%; herbicide
**LAINTRI**	miclobutanil 12.5%; control of *Odium* spp
**MELODY COMBI**	folpet 56,3% + iprovalicarb 9%; control of mildew
**FLINT**	trifloxistrobin 50%; control of *Odium* spp
**GLITAN**	glyphosate; post-emergence herbicide
**2017**<br>**Agent**	**Description**
**ZARPA**	diflufenican 4% + glyphosate 16%; herbicide
**GLITAN**	glyphosate; post-emergence herbicide
**GLIFOSATEC**	glyphosate; post-emergence herbicide
**ACILIQ NPK**	complex fertilizer (inorganic)
**SOLUCION NITROGENADA**	nitrogen fertilizer
**MICROFER CA**	Calcium
**MICROFER MG**	Magnesium
**PROTAMINAL PLUS**	amino acids
**ARMETIL**	folpet 40% + metalaxyl 10%; control of mildew
**MICLOBUTANIL**	miclobutanil 12.5 %; control of *Odium* spp
**VAMECTIN**	abamectina 1.8%; acaricide (pesticide)
**AZUFRE LIQUIDO 80 PALLARES**	sulfur concentrated suspension; control of red spider mite
**COLLIS**	boscalida 20% + kresoxim-metyl 10%; control of *Odium* spp
**DELTAPLAN**	deltametrin 2.5%; control of cluster moth
**KARATHANE STAR**	meptildinocap 35%; control of *Odium* spp
**NOVAZUFRE 98.5%**	sulfur 98.5%, red spider mite
**VIVANDO**	metrafenona 50%; control of *Odium* spp
**GLIMUR GREEN**	glyphosate 36%; post-emergence herbicide

Supplementary Table S4. Chemical data of Negro Saurí grapes and wine for vintages 2014-2017

**Grapes (average data for 15 clones)**									
Vintage	ºBrix	Density	Sugar ($g\ L^{-1}$)	pH	Total acidity ($g\ L^{-1}$)	Malic acid ($g\ L^{-1}$)	Tartaric acid ($g\ L^{-1}$)	Potassium ($mg\ L^{-1}$)	I.P.T.
2014	24.5 ± 0.7	1.1 ± 0.0	244.4 ± 8.6	No data	No data	1.8 ± 0.3	8.0 ± 0.5	1528.7 ± 281.6	12.7 ± 1.6
2015	24.5 ± 1.2	1.1 ± 0.0	244.3 ± 13.8	3.3 ± 0.1	5.72 ± 0.5	2.3 ± 0.6	5.7 ± 0.6	1534.0 ± 143.2	14.7 ± 3.8
2016	24.4 ± 1.5	1.1 ± 0.0	242.8 ± 17.7	3.4 ± 0.1	4.84 ± 0.4	2.0 ± 0.5	4.8 ± 0.4	1594.7 ± 114.7	14.5± 6.7
2017	24.6 ± 0.7	1.1 ± 0.0	245.5 ± 8.6	3.5 ± 0.1	5.98 ± 0.5	2.5 ± 0.3	7.5 ± 0.4	1973.3 ± 98.1	4.3± 0.8

**Wine (from single fermentations)**				
Vintage	Alcohol % (v/v)	pH	Total acidity ($g\ L^{-1}$)	Volatile acidity ($g\ L^{-1}$)
2014	14.12	3.48	4.03	0.36
2015	13.5	3.37	3.9	0.34
2016	13.8	3.55	4.1	0.25
2017	14.5	3.71	3.6	0.25

I.P.T. Total Polyphenol Index (Indice de Polyphénols Totaux)
Total acidity ($g\ L^{-1}$ in sulphuric acid)
Volatile acidity ($g\ L^{-1}$ as acetic acid)
